# The Adaptive COVID-19 Treatment Trial-1 (ACTT-1) in a real-world population: a comparative observational study

**DOI:** 10.1186/s13054-020-03406-3

**Published:** 2020-12-07

**Authors:** Matilde Tejlbo Frost, Espen Jimenez-Solem, Mikkel Zöllner Ankarfeldt, Martin Erik Nyeland, Anne Helms Andreasen, Tonny Studsgaard Petersen

**Affiliations:** 1 Department of Clinical Pharmacology, Copenhagen University Hospital, Bispebjerg and Frederiksberg Hospital, Bispebjerg Bakke 23, indgang 20 C, 2. sal, 2400 Copenhagen, NV Denmark; 2 Department of Clinical Medicine, University of Copenhagen, Copenhagen, Denmark; 3 Copenhagen Phase IV Unit (Phase4CPH), Department of Clinical Pharmacology and Center for Clinical Research and Prevention, Copenhagen University Hospital, Bispebjerg and Frederiksberg, Copenhagen, Denmark; 4 Center for Clinical Research and Prevention, Copenhagen University Hospital, Bispebjerg and Frederiksberg, Copenhagen, Denmark

## Background

The recently published ‘Adaptive COVID-19 Treatment Trial’ (ACTT-1) showed that remdesivir is a promising treatment option against coronavirus disease 2019 (COVID-19) [1]. Consequently, remdesivir is now being evaluated for implementation in clinical practice worldwide.

Randomized clinical trials (RCTs) are the current golden standard for procuring evidence of a drug’s efficacy, but in order to predict effectiveness and safety in daily clinical practice, it is important to complement the results from RCTs with an evaluation of their transferability to a real-world setting.

To bridge the evidentiary gap between clinical research and clinical practice, the U.S. Food and Drug Administration recognizes the need for harnessing ‘Real-World Data’ and observational methods to generate evidence of effectiveness to support regulatory decisions concerning drugs [2].

## Objective

The aim of the present study was to examine whether the evidence generated in the ACTT-1 could be applied to a real-world population by comparing characteristics of the included patients and their outcomes in order to evaluate the transferability of the trial’s outcomes to the patients eligible for remdesivir treatment in clinical practice.

## Methods and findings

Data for the present study were extracted from hospital electronic health records of all patients with a positive severe acute respiratory syndrome coronavirus 2 (SARS-CoV-2) test in the Capital Region of Denmark admitted to a hospital between March 1 and May 5, 2020. Patients’ eligibility was assessed using inclusion and exclusion criteria from ACCT-1. Index time for baseline characteristics and start of follow-up was defined as 24 h after admission or time of first positive SARS-CoV-2 test result, whichever came last based on an assumption that most patients would have been included in the ACCT-1 trial prior to this timepoint. We assessed mortality and time to discharge as a comparable outcome to time to recovery in ACCT-1, during the first 29 days. Indirect standardization was used to weight the cohort to the same eight-point ordinal severity baseline score as the placebo group in the ACTT-1.

**Fig. 1:**
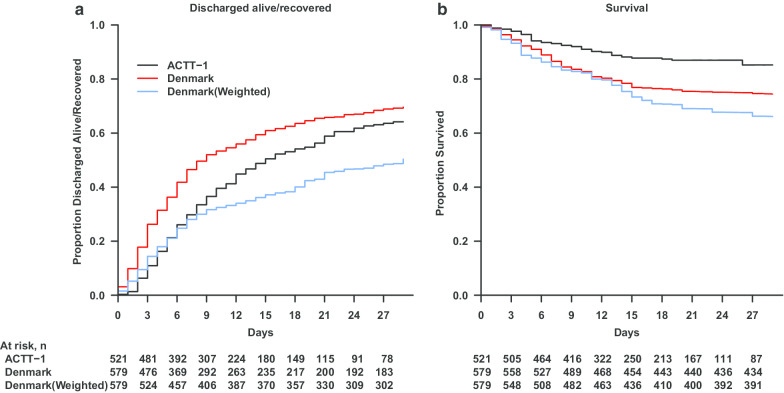
Kaplan–Meier estimates of cumulative recoveries and mortality by 14 days. Estimates of cumulative recoveries (**a**) and mortality by 14 days (**b**) for patients in the Capital Region of Denmark and the placebo group in the ACTT-1 study

We identified 1053 patients admitted with COVID-19. Four hundred and seventy-four patients were ineligible according to inclusion criteria (385 due to mild disease) and exclusion criteria (84 due to severe chronic kidney disease). The remaining 579 patients had complete follow-up. Compared to the placebo group in the ACTT-1, the patients in the present study were older and less obese and fewer required high-flow oxygen, non-invasive ventilation (NIV) or ventilator treatment (Table 1). The overall study population had a shorter duration to discharge and increased mortality compared to the ACTT-1 placebo group (Fig. 1). Adjusting for differences in baseline severity by weighting the study population increased the time to discharge and to a lesser degree mortality. Twenty-two deaths, of 148, occurred after discharge.

**Table 1:** Patient characteristics at baseline and outcomes

Characteristics and outcomes	ACTT-1, placebo group (*n* = 522)	Capital Region of Denmark (*n* = 579)	Capital Region of Denmark, weighted (*n* = 579)
Age (mean, standard deviation, years)	59.2 (15.4)	69.0 (14.9)	67.1 (14.5)
Age intervals (*n*, %)			
18–39 years	60 (11.5)	22 (3.8)	(5.2)
40–64 years	264 (50.6)	174 (30.1)	(30.8)
65 + years	198 (37.9)	383 (66.1)	(64.0)
Male sex (*n*, %)	332 (63.6)	329 (56.8)	(57.7)
BMI (mean, standard deviation, kg/m^2^)	30.5 (7.3)	27.4 (6.4)	28.0 (6.2)
Summary of comorbidities (*n*, %)			
None	102 (22.5)	132 (22.8)	(23.1)
One	117 (25.8)	165 (28.5)	(33.7)
Two or more	234 (51.7)	282 (48.7)	(43.3)
Coexisting comorbidities (*n*, %)			
Hypertension	229 (49.9)	299 (51.6)	(46.0)
Coronary artery disease	46 (10.0)	92 (15.9)	(14.0)
Congestive heart failure	23 (5.0)	51 (8.8)	(7.3)
Chronic respiratory disease (emphysema)	4 (0.9)	92 (15.9)	(16.0)
Asthma	47 (10.3)	44 (7.6)	(8.7)
Chronic liver disease (chronic hepatitis, cirrhosis)	9 (2.0)	12 (2.1)	(1.8)
Chronic kidney disease	22 (4.8)	11 (1.9)	(1.4)
Diabetes (type 1 + 2)	135 (29.6)	133 (23.0)	(22.9)
Obesity	165 (36.2)	129 (27.7)	(27.9)
Cancer	32 (7.0)	81 (14.0)	(12.8)
Immune deficiency (acquired or innate)	36 (7.9)	48 (8.3)	(9.5)
Treatment requirement on hospital admission (*n*, %)*			
Hospitalized, not requiring oxygen	60 (11.9)	141 (24.4)	(11.9)
Hospitalized, requiring oxygen	199 (39.4)	365 (63.0)	(39.4)
Hospitalized, NIV or high-flow oxygen	99 (19.6)	50 (8.6)	(19.6)
Hospitalized, mechanical ventilation or ECMO	147 (29.1)	23 (4.0)	(29.1)
Outcomes, overall			
Median time to recovery/discharged alive in days	15 (13–19)	9 (7–11)	29 (21–NE)
Death day 14, Kaplan–Meier estimate %	11.9 (9.2–15.4)	21.6 (18.2–24.9)	24.6 (18.4–30.6)

*The 17 patients with missing baseline score in ACCT-1 are not included in the denominator

## Discussion

Overall, our study shows that patient characteristics and outcomes in the ACTT-1 differ from the present real-world population. The most pronounced differences are a doubled mortality rate and a larger proportion of patients only requiring supplemental oxygen in the Danish real-world cohort. The increased mortality rate is likely due to the cohorts higher age [3].

In the ACTT-1, the most significant reduction in mortality and an increase in recovery rate were reported for the subgroup of patients only requiring supplemental oxygen. Hence, the observed differences with the present cohort may indicate a potentially larger absolute mortality reduction by remdesivir in a real-world population compared to the ACTT-1, assuming the relative mortality reduction observed in the supplemental oxygen subgroup in the ACTT-1 persists.

Due to the observational nature of the present study, results should be interpreted with caution. We believe, however, that the results are an important supplemental tool to better evaluate the possible impact of introducing remdesivir in clinical practice.

## Data Availability

The datasets generated and/or analyzed during the current study are not publicly available due Danish law restricting the sharing of non-anonymous personal data.
